# Empathy-related abnormalities among women with premenstrual dysphoric disorder: clinical and functional magnetic resonance imaging study

**DOI:** 10.1192/bjo.2024.723

**Published:** 2024-08-05

**Authors:** Yulia Lerner, Gal Raz, Miki Bloch, Michael Krasnoshtein, Michal Tevet, Talma Hendler, Oren Tene

**Affiliations:** Sagol Brain Institute, Tel Aviv Sourasky Medical Center, Israel; Faculty of Medicine, Tel Aviv University, Israel; and Sagol School of Neuroscience, Tel Aviv University, Israel; Sagol Brain Institute, Tel Aviv Sourasky Medical Center, Israel; Sagol School of Neuroscience, Tel Aviv University, Israel; and Steve Tisch School of Film and Television, Faculty of the Arts, Tel Aviv University, Israel; Faculty of Medicine, Tel Aviv University, Israel; Psychiatric Division, Tel Aviv Sourasky Medical Center, Israel; and Brull Ramat Chen Mental Health Center, Tel Aviv, Israel; Faculty of Medicine, Tel Aviv University, Israel; Psychiatric Division, Tel Aviv Sourasky Medical Center, Israel; and Ambulatory Department, Yehuda Abarbanel Mental Health Medical Center, Bat Yam, Israel; Psychiatric Division, Tel Aviv Sourasky Medical Center, Israel; Sagol Brain Institute, Tel Aviv Sourasky Medical Center, Israel; Faculty of Medicine, Tel Aviv University, Israel; Sagol School of Neuroscience, Tel Aviv University, Israel; and School of Psychological Sciences, Tel Aviv University, Israel; Faculty of Medicine, Tel Aviv University, Israel; and Psychiatric Division, Tel Aviv Sourasky Medical Center, Israel

**Keywords:** Mood disorder, naturalistic stimulation, neuroimaging, affective empathy, cognitive empathy

## Abstract

**Background:**

Empathy refers to the cognitive and emotional reactions of an individual to the experiences of another. Women with premenstrual dysphoric disorder (PMDD) report severe social difficulties during the luteal phase of their menstrual cycle.

**Aims:**

This clinical and functional magnetic resonance imaging study aimed to explore affective and cognitive empathy in women with PMDD, during the highly symptomatic luteal phase.

**Method:**

Overall, 32 women with PMDD and 20 healthy controls participated in the study. The neuroimaging data were collected using a highly empathy-engaging movie. First, we characterised the synchrony of neural responses within PMDD and healthy groups, using the inter-individual correlation approach. Next, using network cohesion analysis, we compared connectivity within and between brain networks associated with affective and cognitive empathy between groups, and assessed the association of these network patterns with empathic measures.

**Results:**

A consistent, although complex, picture of empathy abnormalities was found. Patients with PMDD showed decreased neural synchrony in parietal and frontal key nodes of cognitive empathy processing (theory-of-mind network), but higher neural synchrony in the anterior insula and anterior cingulate cortex, a part of the salience network, implicated in affective empathy. Positive correlations between cognitive perspective-taking scores and neural synchrony were found within the theory-of-mind network. Interestingly, during highly emotional moments, the PMDD group showed increased functional connectivity within this network.

**Conclusions:**

Similar to major depression, individuals with PMDD show enhanced affective empathy and reduced cognitive empathy. These findings echo clinical observations reported when women with PMDD have a dysregulated emotional response to negative stimuli.

Most women of reproductive age experience emotion-related premenstrual symptoms, and in up to 8% of women, the severity of the clinical presentation warrants a diagnosis of premenstrual dysphoric disorder (PMDD).^1^ This disorder was officially added to the mood disorders in DSM-5; it is characterised by affective, cognitive, behavioural and somatic symptoms that occur in the luteal phase of the menstrual cycle, and typically resolve soon after the onset of menses.^2^ It is assumed that fluctuations in sex hormone levels affect the emotional state of women with PMDD, increasing the risk of negative dysregulated emotions during the luteal phase and causing the hallmark symptoms of the premenstrual mental suffering, including depressed and/or labile mood, irritability, anger and anxiety.

## Empathic distress in PMDD

Among the affective disturbances related to premenstrual symptoms, poor stress regulation has gained substantial attention. Behavioural studies reported an enhanced reactivity to stress-related stimuli in PMDD, especially during the luteal phase.^3^ This corresponds to growing evidence implicating PMDD with dysregulation of the hypothalamus-pituitary-adrenal axis, which mediates adaptive stress responses.^4^ Unlike stress dysregulation, the notion of heightened empathic distress – a stress response related to another person being in a stressful situation – has been largely overlooked in PMDD. Empathic distress is a core aspect of affective empathy, which refers to a homological vicarious somatic response to another's affective state based on bottom-up afferent signalling and interoception.^5^ It is distinguished from higher-order processes of cognitive empathy, where another's perspective is adopted to facilitate inference of their mental state (including motives, beliefs, goals, etc.). This distinction has been corroborated in the past two decades by numerous neuroimaging studies, suggesting a differential neural basis for these processes.^6,7^ The scientific blind spot about empathic distress in PMDD is peculiar, considering accumulating evidence of the significance of empathic distress in other affective disorders such as major depressive disorder (MDD).

Two meta-analyses^8,9^ found replicable high levels of empathic distress in individuals with MDD compared with controls, and positive correlations between personal distress and depression indices in analogue samples. Interestingly, the literature also points to an opposite pattern of correlation between depression and indices of cognitive empathy.^8,9^ Depression was found to be associated with reduced objective measures of cognitive empathy, as well as with abnormal self-report measures of perspective-taking,^10^ although the latter evidence is weaker. Previous data on empathy-related dissociation suggest that enhanced affective empathy and reduced cognitive empathy results from heightened self-focus processing among individuals with depression;^9^ that is, from increased susceptibility affective processes that give salience to the current state of one's own body, rather than cognitively focusing on the other's perspective and personal concerns.^11^ This insight has clinical significance, as training individuals with MDD to switch to another person's perspective may not only improve social skills, but also directly reduce depressive symptoms such as self-preoccupation.^12^ For such intervention to be more mechanism-based and precise, it should be linked to empathy-related neural processes.

## Neural networks involved in affective and cognitive empathy

The processing of affective empathy has consistently been found to engage a network whose central nodes are the mid/anterior cingulate cortex and the anterior insula, whereas mentalising, a major aspect of cognitive empathy and the theory-of-mind (ToM) network, commonly involves dorsal and ventral aspects of the medial prefrontal cortex (mPFC), temporal parietal junction (TPJ) and superior temporal sulcus.^7^ In PMDD, neuroimaging research vastly focused on neural abnormalities related to affect dysregulation, pointing to dysfunction of the dorsal lateral prefrontal cortex.^13^ However, to the best of our knowledge, none of these studies directly examined possible empathy-related neural abnormalities in PMDD.

To address this gap, we first compared individuals with PMDD with healthy controls, using the Interpersonal Reactivity Index (IRI),^14^ which offers distinct probes for affective empathy of distress and cognitive empathy of perspective-taking. We expected individuals with PMDD to score higher on personal distress and lower on perspective-taking. Next, we conducted a naturalistic neuroimaging experiment, in which we evoked sadness in participants by watching a tragic movie segment (from the movie *Stepmom*, 1998) that depicts the agony and distress of a family when the mother tells her children about her terminal disease. In previous neuroimaging studies, we found evidence that this film excerpt engages different dynamic connectivity patterns of the two brain networks that have been associated with the distinct empathy-related modes indicated above.^15,16^

**Fig. 1:**
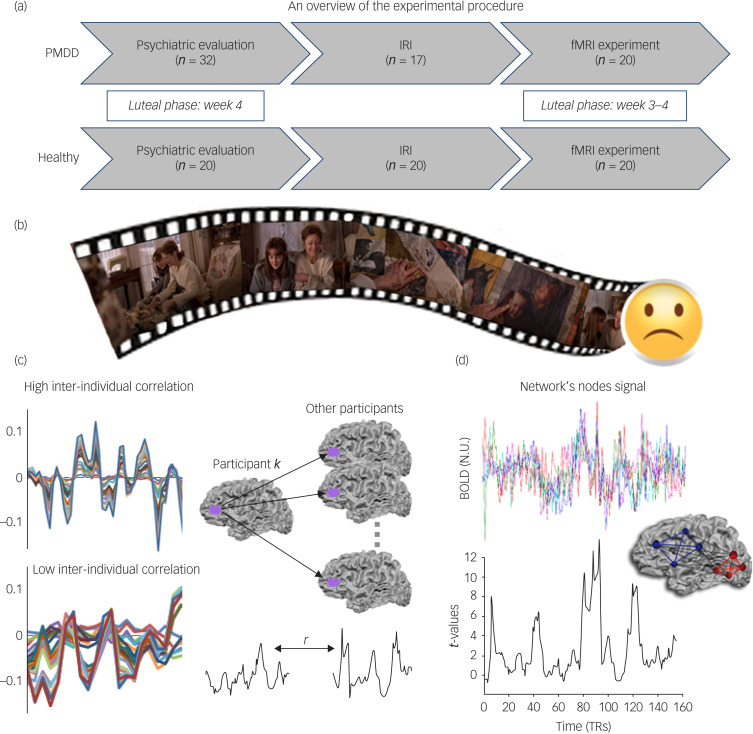
(a) An overview of the experimental procedure. (b) Excerpted sequence from the *Stepmom* movie, which all participants watched during the fMRI session. (c) Inter-individual correlation analysis in brief. The neural responses over the course of the clip duration were extracted from area IFS for both groups of participants (left). A schematic presentation of inter-individual correlation analysis between an individual participant and all other participants (right). (d) Network cohesion analysis in brief. Two schematic networks with four nodes are illustrated in blue and red. Correlation coefficients for edges between nodes within each of the networks (solid coloured lines) are *t*-tested to compute intra-NCI. Inter-NCI is similarly computed for edges between networks (dashed lines). NCI dynamics: NCI is computed in sliding windows of ten repetition times (TRs). The coloured curves represent the BOLD time courses of different nodes within a network in a representative participant. The intra-NCI of this network for the specific participant is presented at the bottom panel. Note that the NCI increases with the local covariance between the signals. BOLD, blood-oxygen-level-dependent; fMRI, functional magnetic resonance imaging; IFS, inferior frontal sulcus; IRI, Interpersonal Reactivity Index; NCI, Network Cohesion Index; PMDD, premenstrual dysphoric disorder.

Our study examined whether PMDD is characterised by a modified pattern of activity and connectivity within empathy-related networks during a naturalistic empathy sadness-provoking cinematic scenario. We addressed this question by using both data- and hypothesis-driven analytical methods (Fig. 1) in participants during the luteal phase. First, we adopted an inter-individual correlation approach^17^ to examine voxel-wise group differences in the patterns of neural responses to the movie stimulus. We then conducted a hypothesis-driven analysis^18^ to investigate whether connectivity within and between brain networks associated with affective and cognitive empathy differs between PMDD and healthy control groups during cinematic moments of high sadness. Finally, we examined the association of such network patterns with empathic distress measures and specific symptoms.

## Method

### Participants

Fifty-two women of reproductive age, with a regular menstrual cycle, participated in the study when in their luteal phase. Of them, 32 women diagnosed as suffering from PMDD (age range 19–46 years; mean age 33.3 ± 6.6 years) were recruited from the Ambulatory Psychiatric Department at the Tel Aviv Sourasky Medical Center (TASMC). Twenty patients with PMDD participated in the functional magnetic resonance imaging (fMRI) experiment, nine patients took part in the behavioural test (IRI) exclusively and eight patients participated in both sessions. Specifically, nine out of 32 patients only underwent the IRI, eight out of 32 patients completed both the fMRI session and the IRI, and 12 out of 32 patients exclusively underwent the fMRI. In addition, 20 healthy women (age range 19–48 years; mean age 32.1 ± 8.0 years) were recruited as a control group. Three additional patients with PMDD were excluded from the analyses because of excessive head motion (>3 mm) and technical problems during the session (image distortion). All participants had normal or corrected vision, no hearing impairment and were right-handed, fluent Hebrew speakers with adequate language comprehension.

The diagnosis of provisional PMDD was established by a senior psychiatrist, based on DSM-5 criteria^1^ and confirmed by prospective follow-up of at least two symptomatic menstrual cycles, using the Daily Record of Severity of Problems.^19^ Ovulation and the subsequent onset of the luteal phase were confirmed by a standard commercial urine test. Exclusion criteria included moderate-to-severe polycystic ovary disease, usage of a hormonal intrauterine device, recent initiation of hormonal or antidepressant treatment (<3 months), or drug misuse other than nicotine in the past 3 months. Patients were free of a major depressive episode or an active anxiety disorder, and without active treatment for these disorders at the time of the study. Control participants were screened using the Premenstrual Symptoms Screening Tool to validate that they did not have PMDD.^20^

All procedures contributing to this work complied with the ethical standards of the institutional committee on human experimentation and with the Helsinki Declaration of 1975, as revised in 2008. All procedures involving human participants/patients were approved by the Ethics Committee for Human Studies at TASMC (approval number TLV-0034-15; ClinicalTrials.gov trial registration number NCT02448836), and all participants provided written informed consent to participate in the study.

### Procedure

Each participant was examined on two separate days, no more than a month apart. On the first meeting, a psychiatric evaluation was conducted, and on the second meeting, a neuroimaging session was performed. The psychiatric evaluations were carried out in the second week of the luteal phase (2 or 3 days before the period started). The neuroimaging sessions were conducted on day 23.6 ± 3.3 of the menstrual cycle in the patient group (the earliest day that the scan was conducted was the 17th day of the cycle), and on day 22.8 ± 2.6 in the healthy group (the earliest day that the scan was conducted was the 18th day of the cycle).

### Psychiatric evaluation

PMDD symptoms were assessed with the Premenstrual Tension Syndrome Observer Rating Scale (PMTS-OR),^21^ which is sensitive to the variation in severity of premenstrual symptoms. In addition, the Clinical Global Impressions scale (CGI^22^) and Big Five Inventory (BFI^23^) were administered.

### Empathy measures

Empathy level was evaluated with the IRI,^14^ a tool that consists of perspective-taking, empathic concern, personal distress and fantasy subscales. The perspective-taking and fantasy scales evaluate cognitive components of empathy, whereas the empathic concern and personal distress scales encompass affective aspects. The IRI components have been compared between groups, using multivariate analysis of variance. Spearman's rank correlation was used to reveal associations between IRI and neural synchrony measures for both cognitive and affective components. We also used Spearman's rank correlation test to compare the IRI subscale scores with network cohesion indices computed separately for the two movie segments at repetition times 13 and 27 that were rated as evoking sadness to at least a moderate extent (see Fig. 7 and below). These measures were compared for the ToM and affective empathy intra-network cohesion indices, and the ToM and affective empathy inter-network cohesion index. Since the overlapping Network Cohesion Index (NCI) windows are not exclusive, we employed false discovery rate (FDR) correction for dependent tests to account for multiple comparisons.^24^

### Imaging procedure

Participants were scanned at TASMC on a Siemens 3 T Scanner (MAGNETOM Prisma, Germany), equipped with a 20-channel head coil. The details of the procedure are described in Supplementary Method 1 available at https://doi.org/10.1192/bjo.2024.723. Participants wore active noise-cancelling, MRI-compatible headphones (OPTOACTIVE) and passively viewed the movie clip (Fig. 1) on a back-projection screen located inside the MRI scanner. The total scanning time was approximately 30 min. Following the scan, outside of the magnet, participants answered questions about their emotional experience in the context of the movie plot.

### Movie task

Participants watched the highly empathy-engaging movie clip (8 min 27 s) extracted from the drama film *Stepmom* (1998). We previously established that this scene, where a terminally ill mother is talking to both of her children separately about her future death, elicits a strong emotional response among participants.^15^ There were 1-min periods of blank screen that preceded and followed the clip, and were discarded from all analyses.

### Data analysis

#### Preprocessing

MRI processing was performed using BrainVoyager QX version 2.8 for Windows (Brain Innovation, Maastricht, The Netherlands; https://www.brainvoyager.com) and in-house software written in MATLAB 2020b for Windows 10 (The MathWorks, Inc., Natick, Massachusetts, USA; https://www.mathworks.com/). Preprocessing of the functional data included three-dimensional motion correction, temporal smoothing using a linear trend removal with a high-pass filter, and spatial smoothing with a Gaussian filter (for details see Supplementary Method 2).

#### Neural synchrony within a group

Neural synchrony across participants was evaluated with the inter-individual correlation approach. The method does not require a predefined model of the hemodynamic response function and is relatively insensitive to individual responses.^25^ Thus, brain regions with high inter-individual correlation are interpreted to be involved in stereotyped processing of the stimulus across individuals, whereas regions showing low inter-individual correlation may reflect either weak involvement in processing or idiosyncratic processing across individuals.

The inter-individual correlations within each group were computed on a voxel-by-voxel basis by comparing the time course of the blood-oxygen-level-dependent (BOLD) signal for each voxel for a single participant with the average time course of the other participants in the same group.^17^ First, inter-individual correlations were computed for each individual in each voxel, using a leave-one-out approach. This produced a map of inter-individual correlations in each voxel for each participant separately, in each group. Next, these within-group maps were assessed for statistical significance, using a phase-randomisation procedure, similar to our previous studies.^26^ Briefly, we applied a fast Fourier transformation to the time series, randomised the phase of each Fourier component and inverted the Fourier transformation. The phase randomisation was performed at each iteration (approximately 175 000) of the resampling procedure, before computing the inter-individual correlation. Following each iteration of the permutation test, the maximum correlation value across all voxels was aggregated into a null distribution of maximal correlation values, which controls the family-wise error rate (for review, see Nastase et al^25^).

#### Comparison between groups

The differences in neural synchrony between the PMDD and control groups were assessed with a two-tailed *t*-test within each voxel that exhibited a significant inter-individual correlation value in at least one of the groups. These clusters were defined as regions of interest (ROIs).

#### Network cohesion analysis

To assess differences between groups in the connectivity of networks associated with affective empathy and ToM (Supplementary Table 1), we employed network cohesion analysis.^18,27^ In short, this method probes connectivity within a network as a *t*-statistic over Fisher *Z*-transformed Pearson coefficients for all pairwise correlations between the time courses of its nodes. Similar to this computation of intra-NCI, inter-NCI was computed over all edges connecting couples of nodes of exclusive networks (for details, see Supplementary Method 3). The nodes of the affective empathy- and ToM-related networks were defined based on meta-analyses of empathy for pain^28^ and ToM tasks.^29^

#### Correlation with symptoms

A correlation value between the patient's time course and the computed average time course of the rest patients was used as a measure of patient synchrony. This measure was correlated with each symptom (depression, anxiety, etc.) and BFI personality variables separately. The obtained correlations provided us with a region's ‘association’ with the symptom. To assess the significance of the obtained correlations we computed the null distribution of correlations by repeating the above procedure with phase-shuffled time courses.^26^

Similarly, PMDD symptoms were correlated with network cohesion indices by using Spearman's rank correlation test in the two movie segments at repetition times 13 and 27 that were rated as evoking sadness to at least a moderate extent (Fig. 7). FDR correction was applied to control for multiple comparisons.

Statistical analyses and visualisations were performed and constructed with IBM SPSS Statistics version 24.0 for Windows (IBM Corp., Armonk, New York, USA) and R version 4.0.3 for Windows (R Core Team, Vienna, Austria; https://www.npackd.org/p/r/4.0.3).

## Results

The neuroimaging findings are presented in two sections corresponding to the two types of analyses (inter-individual correlation and NCI) performed in the study. Fig. 1 provides an overview of the experimental procedure (Fig. 1(a)), a picture from the movie clip used as a stimulus (Fig. 1(b)) and a graphic illustration of the two analytical approaches – an example of synchronised time courses of BOLD activation (Fig. 1(c)) and NCI (Fig. 1(d)) within PMDD and control groups. Tables 1 and 2 summarise the basic characteristics of the participants, including sociodemographic details, clinical characteristics and syndrome assessment scores. No differences were found between groups in all tested sociodemographic characteristics. Age and years of education were compared with the Kolmogorov–Smirnoff test, since within-group distributions for some parameters (e.g. age) were not normal; other differences between groups were evaluated by the Fisher exact test.

**Table 1:** Sociodemographic characteristics of individuals who participated in the study

	Patients with PMDD (*n* = 30)	Healthy controls (*n* = 20)	Between-group comparison^a^ *P*-value
Age (years)	33.3 ± 6.6	32.1 ± 8.0	*P* = 0.5 [Kolmogorov–Smirnoff test]
Education (years)	15.1 ± 2.8	17.5 ± 2.8	*P* = 0.4 [Kolmogorov–Smirnoff test]
Marital status (in relationship/not)	15/15	13/7	*P* = 0.5 [Fisher exact test]
Children (yes/no)	12/18	6/14	*P* = 1.0 [Fisher exact test]
Employment (yes/no)	25/5	20/0	*P* = 0.1 [Fisher exact test]

a. Between-group comparison has been performed between a group of patients with premenstrual dysphoric disorder (PMDD) who participated in the functional magnetic resonance imaging scan (*n* = 20) and a group of healthy controls (*n* = 20).

**Table 2:** Clinical characteristics for the patients with premenstrual dysphoric disorder

Clinical characteristics	Patients
Symptoms (years)	11.1 ± 6.9
Menarche age (years)	12.8 ± 1.6
Oral contraception (yes/no)	18/12
Physical training (yes/no)	14/16
Current pharmacological treatment (except psychiatric) (yes/no)	8/22
Psychiatric history	
Psychiatric symptoms (yes/no)	17/13
Psychiatric pharmacology in past (yes/no)	16/14
Current psychiatric pharmacology (yes/no)	6/24
Current psychological treatment (yes/no)	14/16

	Depression	Anxiety	Lability	Anger	Total
Premenstrual Tension Syndrome Observer Rating Scale	3.5 ± 0.5	3.4 ± 0.7	3.7 ± 0.5	3.3 ± 0.5	34.0 ± 4.2

	Extraversion	Neuroticism	Agreeableness	Conscientiousness	Openness to experience
Big Five Inventory	27.4 ± 4.8	25.8 ± 4.4	34.1 ± 4.3	31.1 ± 4.0	38.9 ± 5.9
Clinical Global Impressions Scale			5.2 ± 0.4		

**Fig. 2:**
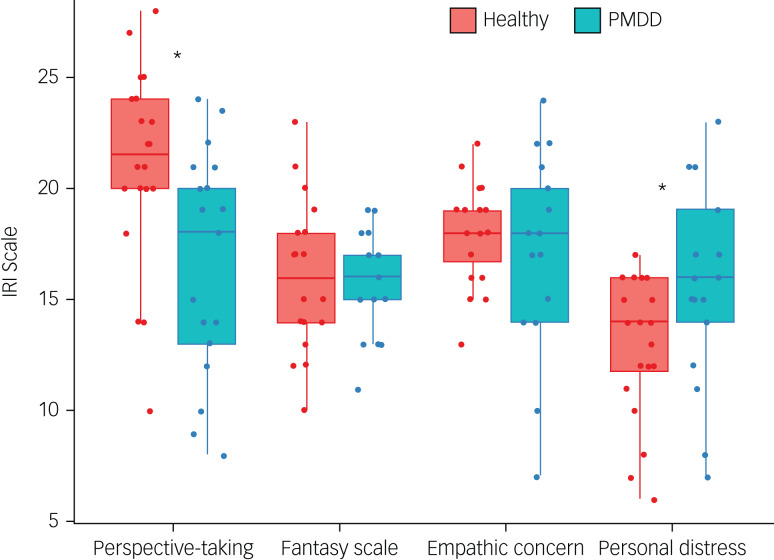
IRI results presenting for patients with PMDD and control participants. Patients with PMDD exhibited a significant deficit in cognitive empathy, specifically in ‘perspective taking’ (*P* < 0.01), but a significantly stronger level of ‘personal distress’, a component of affective empathy (*P* < 0.01). IRI, Interpersonal Reactivity Index; PMDD, premenstrual dysphoric disorder.

The multivariate analysis of variance revealed significant differences between groups in the IRI scores (Fig. 2). Patients with PMDD showed lower scores of cognitive empathy compared with healthy controls, specifically in perspective taking (*P* < 0.01), yet demonstrated significantly stronger personal distress, a component of affective empathy (*P* < 0.01).

### Reliability in neural responses within groups

To evaluate the consistency of the response time courses within each group, we examined the reliability of the neural profiles across individuals within each group. This data-driven approach was used to assess whether individuals within the same group exhibited similar neural response profiles (inter-individual correlation), and the extent to which this was true within the different groups. This approach has been successfully employed in previous studies,^17^ and shows the extent to which the neural responses are synchronised across all locations in the brain.

**Fig. 3:**
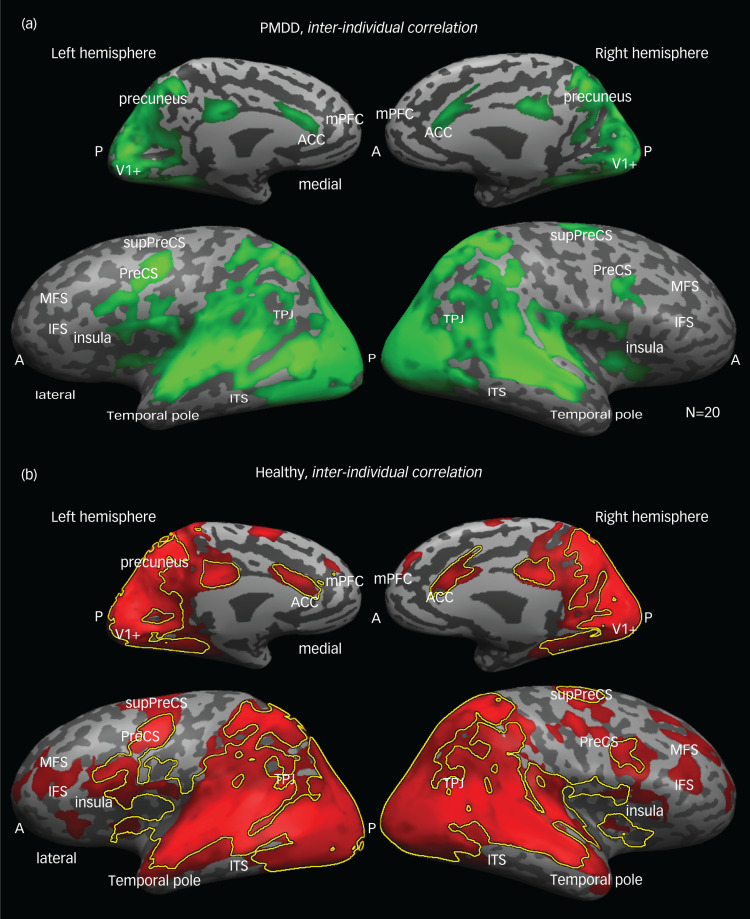
Neural coherence within each group. The maps illustrate the voxels that exhibited neural coherence, or the consistency in response profile across participants within a group during a movie presentation. The analyses were conducted with inter-individual correlation on a voxel-wise basis, and significance was assessed by using a multiple comparisons corrected phase randomisation procedure. (a) Patients with PMDD and (b) healthy controls all exhibited coherence in responses bilaterally throughout the ventral visual pathway, posterior parietal cortex, and full extent of the middle and superior temporal gyri on the lateral surface, and medially in the precuneus and anterior cingulate gyrus. The yellow line shows a border of the ‘PMDD map’ overlaying the ‘healthy map’, indicating that most responses in the posterior, parietal and temporal areas were similar between groups. Patients exhibited less coherent response than controls in the medial prefrontal cortex (mPFC), superior temporal sulci, temporal parietal junction (TPJ), temporal poles and precuneus. Additional regions that showed higher inter-individual correlations in the control group are the inferior and middle frontal sulci (IFS and MFS) and the precentral sulcus (PreCS). A and P refer to the anterior and posterior parts of the brain, and many cortical landmarks are labelled for convenience of viewing. ACC, anterior cingulate cortex; PMDD, premenstrual dysphoric disorder.

Fig. 3 shows the voxel-by-voxel inter-individual correlation maps across the whole cortex within each group (PMDD (Fig. 3(a)) and healthy controls (Fig. 3(b)), revealing the brain areas that responded similarly to the movie across participants within a given group. Visual inspection of the maps reveals that both groups demonstrated high synchronisation of neural responses in the visual- and auditory-related areas. High inter-individual correlation values were found throughout the length of the middle and superior temporal sulci into the inferior parietal lobule and precuneus. Specifically, highly synchronised neural responses were observed in visually related parts of the posterior cortex, including retinotopic and higher-order visual areas, auditory-related areas and areas involved in content and linguistic processing (superior temporal sulcus, angular and supramarginal gyri, precuneus, inferior occipital gyrus).

### Comparison of neural responses between groups

**Fig. 4:**
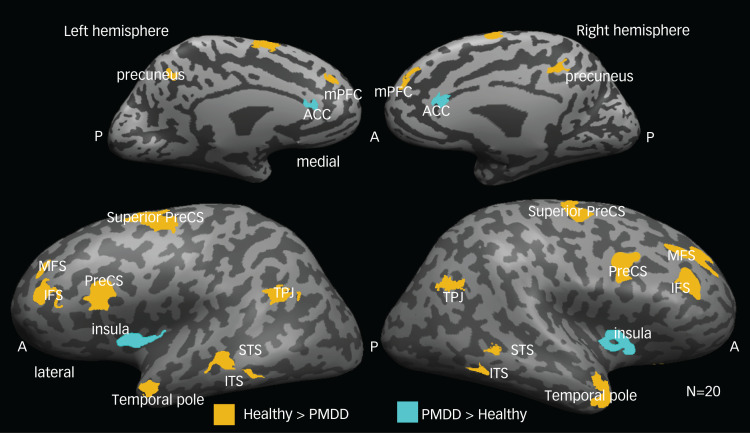
Comparing neural coherence between groups during movie presentation. Significant differences between responses of healthy participants and patients with PMDD (two-tailed *t*-test). Yellow-coloured voxels present regions of interest where inter-individual correlations were significantly higher in the healthy group than in the PMDD group; cyan-coloured regions show the opposite result. ACC, anterior cingulate cortex; IFS, inferior frontal sulcus; MFS, middle frontal sulcus; mPFC, medial prefrontal cortex; PMDD, premenstrual dysphoric disorder; PreCS, precentral sulcus; STS, superior temporal sulcus; TPJ, temporal parietal junction.

A direct comparison of the inter-individual correlation maps per group presented in Fig. 3 exhibited high overlap between them (see yellow line in Fig. 3(b) showing a border of the ‘PMDD map’ overlaying the ‘healthy map’), indicating that most responses in the posterior, parietal and temporal areas were similar between healthy participants and patients with PMDD. However, several regions showed higher inter-individual correlation in the control relative to the PMDD group. Strikingly, most of these regions have been previously consistently associated with the default mode network (DMN), which is known to be involved in cognitive empathy, including mentalisation: the mPFC, superior temporal sulci, TPJ, temporal poles and precuneus. Additional regions that showed higher inter-individual correlation in the control group are the inferior and middle frontal sulci and the precentral sulcus. Note that not all of these non-overlapped regions were significantly different between the groups on a direct comparison. Fig. 4 shows regions that exhibited significant differences (two-tailed *t*-test) when comparing results in the healthy controls and PMDD groups (per ROI). Importantly, two regions bilaterally exhibited significantly higher inter-individual correlations in the PMDD group: the anterior insula and the anterior cingulate cortex, which are the key nodes of the affective empathy network. Table 3 provides the Talairach coordinates and cluster information for these ROIs.

**Table 3:** Talairach coordinates of the brain regions exhibiting the significant difference in between-group comparison

		Talairach coordinates			
Brain region	Brodmann area	*x*	*y*	*z*	Cluster size
Healthy > PMDD					
Frontal lobe					
Left middle frontal sulcus (MFS)	9	−32	37	29	2137
Right middle frontal sulcus (MFS)	9	31	37	33	1000
Left inferior frontal sulcus (IFS)	44	−46	11	14	3267
Right inferior frontal sulcus (IFS)	9	47	15	23	2788
Left precentral sulcus (PreCS)	44	−46	12	12	1515
Right precentral sulcus (PreCS)	44	47	10	11	1391
Left superior precentral sulcus (superior PreCS)	6	−27	−10	55	2000
Right superior precentral sulcus (superior PreCS)	6	29	−11	54	2100
Left medial prefrontal cortex (mPFC)	8	−6	44	39	1177
Right medial prefrontal cortex (mPFC)	8	7	45	40	1964
Parietal lobe					
Left precuneus	7	−8	−54	42	715
Right precuneus	7	4	−53	38	953
Temporal lobe					
Left temporal parietal junction (TPJ)	39	−47	−59	34	1325
Right temporal parietal junction (TPJ)	39	44	−57	35	1645
Left superior temporal sulcus (STS)	21	−58	−18	−1	2295
Right superior temporal sulcus (STS)	41	55	−20	4	1693
Left inferior temporal sulcus (ITS)	20	−52	−48	−9	893
Right inferior temporal sulcus (ITS)	20	52	−47	−11	840
Left temporal pole/superior temporal gyrus	38	−44	9	−25	540
Right temporal pole/superior temporal gyrus	38	43	10	−24	894
PMDD > healthy					
Insular cortex					
Left insula	13	−36	11	8	1376
Right insula	13	41	11	4	1410
Cingulate cortex					
Anterior cingulate	32	0	21	30	573

PMDD, premenstrual dysphoric disorder.

### Relationship between the empathy trait measurements and neural synchrony

**Fig. 5:**
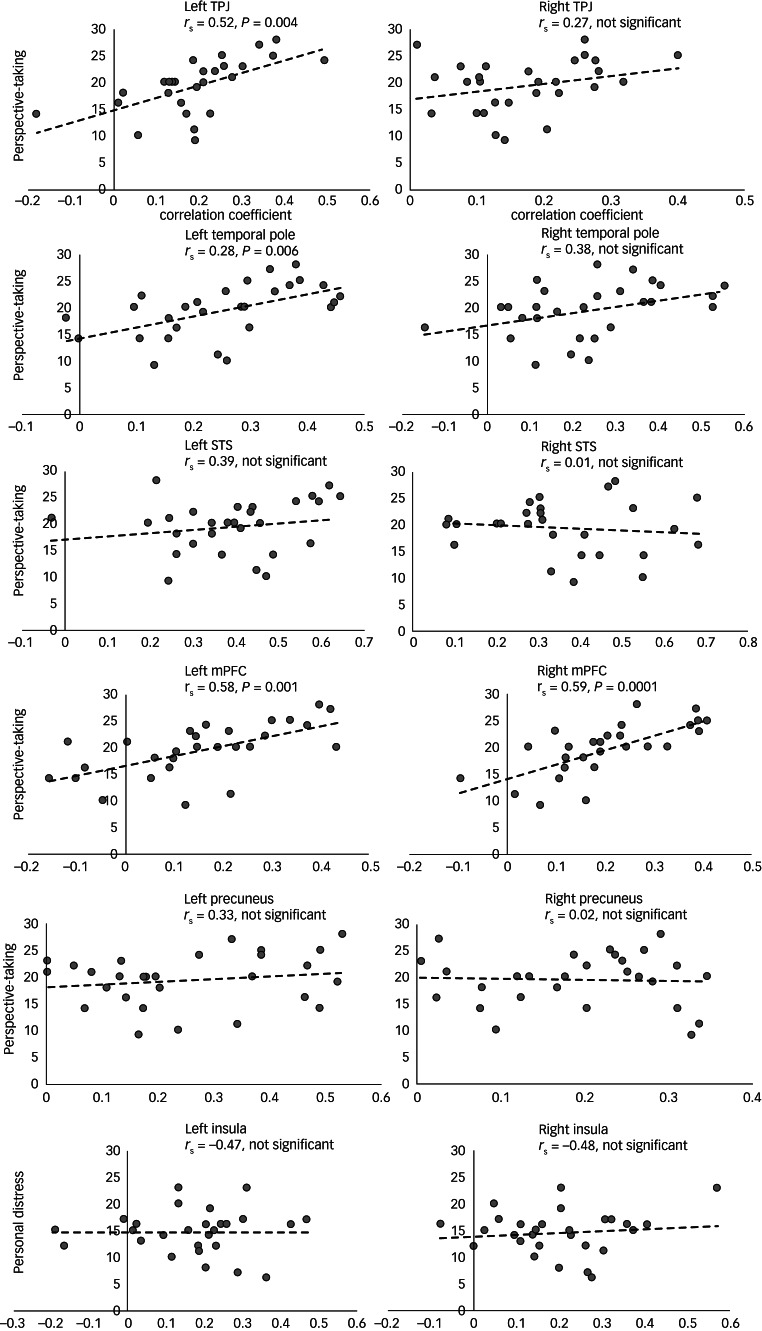
Association between the empathy scales and reliability of brain responses. Correlation between IRI components and inter-individual correlation values computed between each patient's time course and the average time course of the rest participants. Significant correlation between the perspective-taking and inter-individual correlation values was found in the left TPJ, left temporal pole and left/right mPFC (Spearman's rank correlation). No other significant IRI associations have been found. IRI, Interpersonal Reactivity Index; mPFC, medial prefrontal cortex; STS, superior temporal sulcus; TPJ, temporal parietal junction.

To check whether synchrony of neural responses in the regions that exhibited significant difference between PMDD and control groups (see Fig. 4) was associated with empathy trait measurements, we computed correlations between IRI components (perspective-taking, empathic concern, personal distress and fantasy scale) and inter-individual correlation values per region of difference. Results presented in Fig. 5 demonstrated a significant correlation (Spearman's rank correlation) between the perspective-taking (a cognitive empathy component), and inter-individual correlation values in the left TPJ (*P* = 0.004, *r*_S_ = 0.52), left temporal pole (*P* = 0.006, *r*_S_ = 0.28) and left/right mPFC (*P* = 0.001, *r*_S_ = 0.58/0.59). No other significant IRI associations were found.

### Relationship between symptom severity and neural measures

**Fig. 6:**
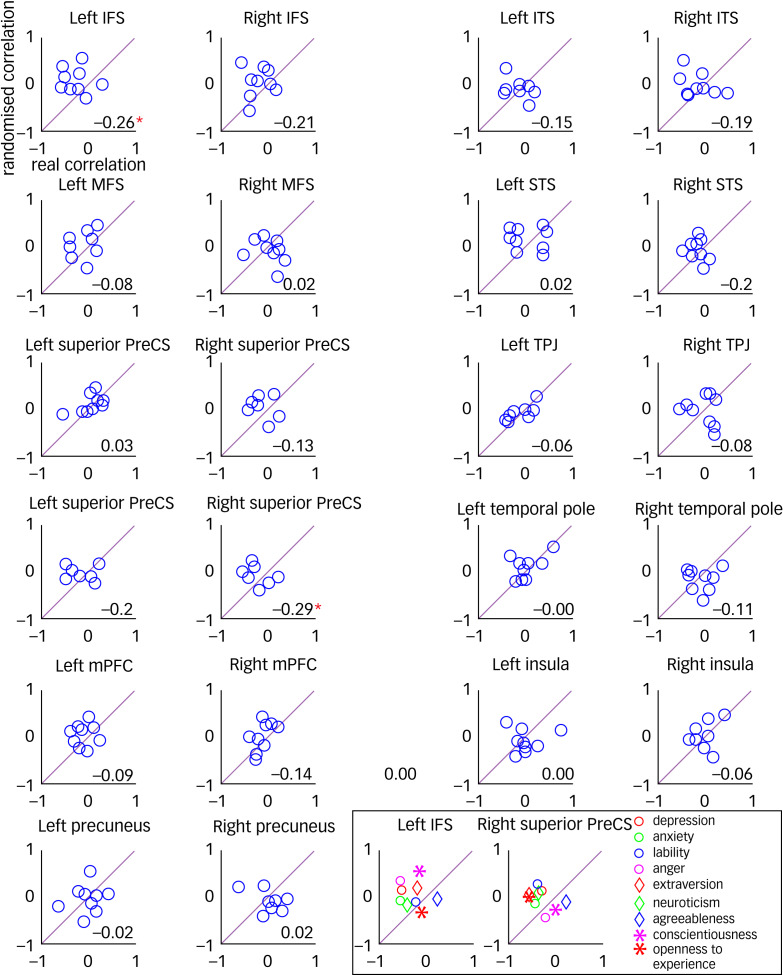
Association between the symptoms (e.g. depression, anxiety, lability, etc.) strength and reliability of brain responses. To check the significance of the coherence measure, we performed a phase randomisation procedure (see Method section). Scatter plots for the mean coherence for each symptom in each ROI measured in the experiment (*x*-axis) and obtained from the shuffled time courses (representing null distribution, *y*-axis) are shown. A cluster of circles in the significantly affected regions (marked by asterisks) – left IFS, right superior PreCS – shifted left of the diagonal line showing smaller average correlations for most symptoms in this region. The numbers represent correlation coefficients between symptom ratings and patient time course coherence. The negative values in significant ROIs indicate that time courses for patients with higher symptom ratings are more deviant than for patients with lower symptom ratings. Some of the ROIs exhibited consistently lower coherence measures concerning all symptoms. An insert shows results for all symptoms separately in the significantly affected ROIs. IFS, inferior frontal sulcus; ITS, inferior temporal sulcus; MFS, middle frontal sulcus; mPFC, medial prefrontal cortex; PMDD, premenstrual dysphoric disorder; PreCS, precentral sulcus; ROI, region of interest; STS, superior temporal sulcus; TPJ, temporal parietal junction.

We further checked whether neural synchrony in the regions that exhibited significant difference between healthy and PMDD groups was associated with symptom severity among patients with PMDD, by computing correlations between rating of symptoms (e.g. depression, anxiety, lability, etc.) and inter-individual correlation values. To check the significance of the correlation measures, we performed a phase randomisation procedure (see Method section). This analysis revealed that some of the ROIs show consistently lower correlation measures concerning all symptoms. Fig. 6 presents scatter plots for the mean correlation for each symptom in each ROI measured in the experiment (*x*-axis) and obtained from the shuffled time courses (representing null distribution, *y*-axis). One can observe that a cluster of circles in the significantly affected regions (left inferior frontal sulcus, right superior precentral sulcus, marked by asterisks) shifted left of the diagonal line, showing smaller average correlations for most symptoms in this region. The numbers in Fig. 6 represent correlation coefficients between symptom ratings and patient time-course coherence. The negative values in significant ROIs indicate that time courses for patients with higher symptom ratings are more deviant than for patients with lower symptom ratings. An insert in Fig. 6 demonstrates results for all symptoms separately in the significantly affected ROIs.

No significant correlations were found between the IRI scores and PMDD symptoms and ToM, affective empathy and ToM–affective empathy NCIs either in the first or the second emotional epochs.

### Group differences in empathy-related network cohesion dynamics

**Fig. 7:**
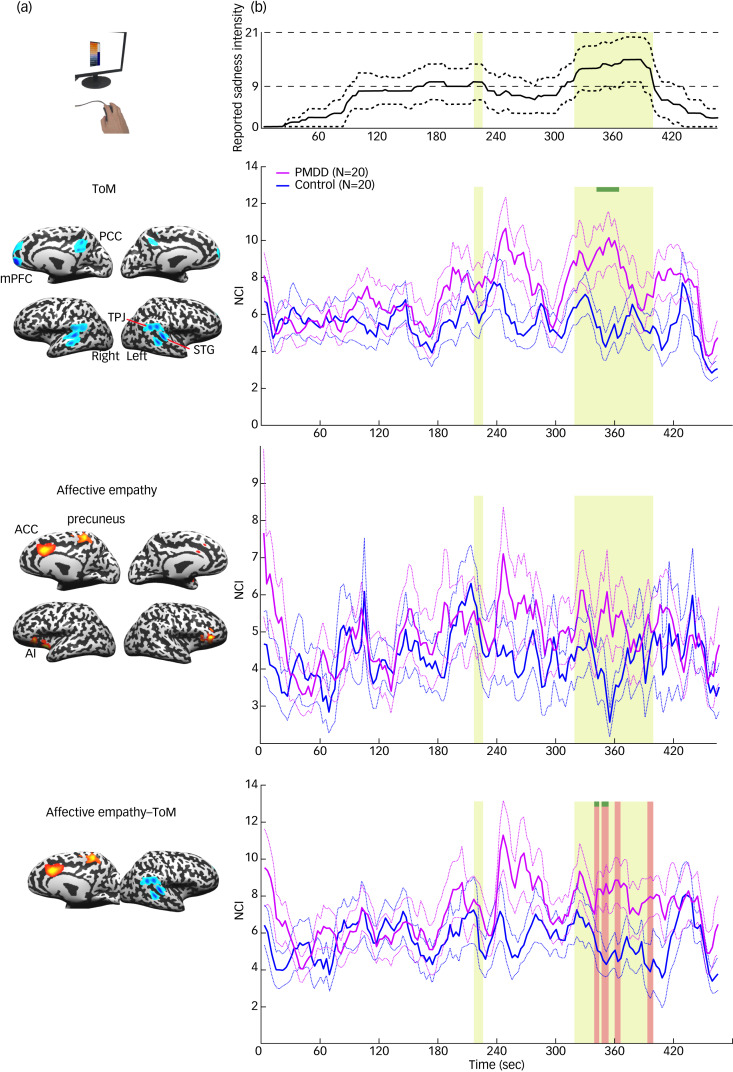
(a) Nodes of the ToM and affective empathy networks. The thresholded maps were obtained from Lamm et al^28^ and Bzdok et al.^29^ (b) Network cohesion dynamics during the viewing of an emotional clip from *Stepmom.* The upper panel presents the median (solid line) and the median absolute deviation from the median (dashed line) of the rating of the sadness intensity by a separate group (*n* = 136). The yellow surface indicates epochs where the median rating corresponded to at least a moderate level of sadness. The other panels illustrate time courses of the mean (solid line) and s.e. (dashed line) of ToM and affective empathy intra-NCI and affective empathy and ToM inter-NCI. The pink surfaces indicate epochs of significant difference between the groups (*Q*_FDR_ < 0.05). Green surfaces at the top of the pink surfaces indicate epochs of spatial specificity higher than 95%. ACC, anterior cingulate cortex; FDR, false discovery rate; mPFC, medial prefrontal cortex; NCI, Network Cohesion Index; PCC, posterior cingulate cortex; PMDD, premenstrual dysphoric disorder; STG, superior temporal gyrus; ToM, theory of mind; TPJ, temporal parietal junction.

Finally, to further elucidate the empathic distress processing, we conducted a hypothesis-driven analysis to examine differences between the groups in the cohesion-related analysis of networks implicated in affective and cognitive empathy. Fig. 7 illustrates the results of this analysis. The general pattern of ToM intra-NCI is similar across groups, except for temporally local differences. A clear and significant difference between the groups appears before the global peak of reported sadness. This cinematic event depicts a mother's highly emotional conversation with her daughter about her future death. It starts with the culmination of both sadness rating and ToM intra-NCI (we previously found that these indices correlate).^15^ However, although in the control group, the ToM NCI decreased after initial ascent, the PMDD group maintained relatively high levels of ToM NCI (see the pink rectangles in Fig. 7(b)). This difference disappeared just before the movie's emotional peak. The significant effect lasted nine consecutive time windows (*Z* ranged between 2.5 and 3.15, *Q*_FDR_ < 0.05). The spatial specificity of these effects was higher than 95% in eight of these windows, ranging from 96 to 99%. This finding indicates that the ToM NCI difference within this epoch cannot be explained as reflecting a whole-brain effect.

Fig. 7 shows that no significant differences between the PMDD and control groups were found when affective empathy intra-NCI was examined. However, in six time windows, higher ToM–affective empathy inter-NCI in the PMDD relative to the control group (*Z* ranged between 2.67 and 3.04, *Q*_FDR_ < 0.05) was observed. Like the ToM NCI, the effect appeared after the rise of both NCI and rating. Spatial specificity higher than 95% (95.5–98.1%) was found in the first three windows.

### Control of the possible treatment-related confounders

To check whether the pharmacological treatment that some patients with PMDD received during the study affected our results, we repeated the analyses on the subgroup of patients. Since the number of patients who received psychiatric medication was small, we were not able to compare the subgroups (with and without medication). Instead, we excluded these patients from the analyses and repeated the inter-individual correlation and NCI analyses, as well as the IRI and behavioural tests, on the subgroup of patients who did not receive pharmacological treatment. A comparison between the results for this subgroup and outcomes for the initial group did not reveal significant differences (Supplementary Appendix 1).

Next, to control for the potential effect of the psychological treatment on the outcomes, we divided the initial group of patients into two subgroups (with and without psychological treatment) and performed the analyses once more. Results are presented in Supplementary Appendix 2. Again, we did not find any significant difference between the tested subgroups, although the number of participants in each subgroup was small.

## Discussion

In this study, we report a consistent picture of empathy abnormalities in individuals with PMDD during the luteal phase of the menstrual cycle. It is important to note that the medical history of nearly half of our patients indicated symptoms of depression and anxiety. Specifically, part of the patients reported mood disturbances that interfered with their everyday lives in the past, and some of them even received medication treatment for this. However, clinical assessment at the time of entry into the study did not reveal a major depressive episode in any of the participants. It can be assumed that at least some of the symptoms described in the past were part of the PMDD, which was not diagnosed previously.

Similar to depression,^9^ individuals with PMDD were scored higher than healthy participants on personal distress scales of the IRI, as well as showing lower levels of perspective-taking relative to the control group. These findings suggest that despite some differences in the symptoms of MDD and PMDD, PMDD similarly involves enhanced affective empathy for distress as well as reduced cognitive empathy, as indicated by the lower capability to adopt another's perspective.

Our data-driven analysis revealed that individuals with PMDD showed higher neural synchrony across participants in the anterior insula/inferior frontal sulcus (bilaterally), and the anterior cingulate cortex when watching a naturalistic depiction of others’ distress (Fig. 4, cyan regions). This remarkably localised pattern of neural response overlaps with the core of the resting state salience network,^30^ which has been implicated in interoception and affective empathy.^6,7^ Surprisingly, the PMDD group also showed decreased neural synchrony in all parietal and frontal key nodes of the ToM-related network, which is considered part of cognitive empathy processing (Fig. 4, yellow).^6,7^ Furthermore, the link between cognitive perspective-taking and neural synchrony within this network during viewing of the specific emotional movie is supported by the positive correlation between the IRI scores and the left TPJ, left temporal pole and mPFC, which are core regions of the ToM (Fig. 5).

Together, these findings suggest that the experience of others’ distress in individuals with PMDD is dominated by affective empathy, where one's affective state is at some level indistinguishable from the other's state.^28^ Yet, as indicated by the IRI-neural correlations, the experience of others’ distress in PMDD may be guided to a smaller degree by cues for cognitive perspective shifts and ToM-related processing, which rely on the maintenance of a separate cognitive representation of the other. This interpretation is in line with the notion that PMDD (at least in the luteal phase), like MDD, involves a self-focused bias, which is manifested as a tendency to allocate attentional resources to self-related processing, mainly somatic and affective, also during social interactions and empathic situations.^9^ Interestingly, self-focused attention is enhanced during the luteal compared to follicular phases, and in women with a premenstrual disorder relative to controls.^31^ This possibility points to a potential intervention strategy in PMDD, in which the individual is trained to engage in other-focused perspective-taking during social interactions.^9^ Such emotion regulation method was already found to reduce stress and depression symptoms in MDD.^12^

At first glance, our NCI and inter-individual correlation findings seem to contradict. Although the PMDD group showed reduced inter-individual correlation in ToM-related areas (Figs 3–4) throughout the movie, the ToM NCI was higher among individuals with PMDD during the early phase of the movie's emotional peak (Fig. 7). However, a careful account of these measures suggests that both these findings can be explained. Inter-individual correlation indicates the extent to which the local neural activity is synchronized across individuals by an external stimulus.^17^ NCI, on the other hand, is a measure of the functional connectivity of a network.^27^ Thus, it is possible that although the activity dynamics of the ToM network are less strictly and consistently dictated by the movie events in the PMDD group, at some point this network will react in ways that are unrelated to the content. The ToM network significantly overlaps with the DMN of regions that have been consistently more activated during rest relative to active task performance.^32^ Importantly, DMN regions have been implicated not only in other-directed cognition, but also in high-level self-referential cognition.^33^ According to a common explanation for this convergence, both high-level self-referential cognition and mentalising require the construction of mental representation that draws on processes of autobiographical memory, prospection and mental simulation; all involved in ToM-related processing.

Thus, the combination of a decreased ToM-related synchrony among participants and increased ToM-related network cohesion may reflect abnormalities in self-referential cognition, which is first triggered by the stimulus, but is next activated according to the individual idiosyncratic line of thoughts. Indeed, in a previous experiment, in which the same movie was displayed to the participants twice, regions within the DMN/ToM network showed activity patterns that were decoupled from external stimulation, unlike sensory areas.^34^

In the context of our study, a potential source for such idiosyncratic processing may be rumination,^35^ which is considered as a form of depressive self-focused attention. It has been moderately associated with depression^36^ and is strongly related to premenstrual disorders.^37^ Interestingly, abnormality in DMN function has been suggested to mediate rumination in depression. For example, individuals with depression failed to reduce activity in DMN regions, including the mPFC, when reappraising negative stimuli after perceiving them.^38^

Thus, it is possible that the enhanced ToM-related network coherence and decreased ToM-related synchrony in the PMDD group are complementary rather than contradictory in pointing to a common underlying process such as emotion regulation deficiency. Although the naturalistic sadness-inducing event at the beginning of the emotional episode (a dialogue between a girl and her dying mother) invoked increased coherence in both groups and each network, the connectivity within these networks subsided in the control, but not in the PMDD group (see Fig. 7, around 300–400 s). This neural pattern might indicate engagement in idiosyncratic rumination among patients with PMDD. Affective empathy–ToM inter-network coherence, which also increased in the PMDD group around the same time, may mediate between the affective embodiment and cognitive aspects of self-focused processing within this group. However, surprisingly, affective empathy-related network coherence did not differ between the groups, whereas neural synchrony did. It is possible that the low temporal resolution (30 s) of the sliding window applied for the analysis of NCI is not sensitive enough to capture differences in the connectivity response to brief affective cues (although it may be sufficient to probe slower processes such as rumination).

Our study demarcates a neural mechanism that could explain the behavioural findings observed in PMDD. It relies on a unique methodological approach that sets it apart from other works, and has the potential to advance our understanding of this common mood disorder at both the basic and clinical level. A deeper understanding of PMDD may help to establish preventive or therapeutic tools to better guide emotional regulation training, and potentially neuromodulation intervention. As computer- and internet-based platforms for PMDD are yet to be developed, our findings suggest a potential direction for non-pharmacological interventions aimed at reducing negative premenstrual symptoms and improving affective conditions. Attention training, which aims to direct attention from self-focused processing toward metacognition,^39^ has shown efficacy in treating depressive disorders.^40^ Our findings suggest that exercising attention shifts not only in the auditory domain, but also those related to affective audio-visual cues provided by moving images may amplify clinical outcomes. Real-time probing of affect sharing and ToM-related networks could further increase the specificity and efficacy of the treatment when integrated into a brain–computer interface.

The main limitation of our study is the relatively small sample of women, yet achieving significant results in this setting strengthens our findings. We also used only a single movie that theoretically does not have a similar effect on different women; nevertheless, previous studies have shown the *Stepmom* movie to be highly effective in causing an emotional response among a broad range of patients. Also, results could be affected by some additional factors like pharmacological and/or psychological treatment, or using oral contraceptives. We have not explored these questions deeply because of the limited number of participants who received the treatment. However, analyses performed on the available subgroups did not reveal significant differences in any of the tests (see Supplementary Appendices 1 and 2). Future studies are required to understand these factors. Finally, we scanned patients with PMDD only in their luteal phase, so their results could not be generalised to other phases of their menstrual cycle. Yet, as symptoms of PMDD occur during this phase, it is at the centre of interest.

Further studies are required to better explain the clinical phenomenon observed in our research, and possibly suggest targets for therapeutical interventions.

## Supporting information

Lerner et al. supplementary material 1 Lerner et al. supplementary material

Lerner et al. supplementary material 2 Lerner et al. supplementary material

Lerner et al. supplementary material 3 Lerner et al. supplementary material

## Data Availability

The unidentified data (e.g. data spreadsheet) that support the findings of this study are available on request from the corresponding author (Y.L.). The data are not publicly available due to medical confidentiality and since participants did not consent to having their data publicly published.
