# Supplementary material for: Empathy-related abnormalities among women with premenstrual dysphoric disorder: clinical and functional magnetic resonance imaging study
Source: BJPsych Open. 2024 Aug 5;10(5):e138. doi: 10.1192/bjo.2024.723 (PMC11698146; doi:10.1192/bjo.2024.723)
Supplement: Lerner et al. supplementary material 1 — Lerner et al. supplementary material [file S2056472424007233sup001.pdf]

Supplementary Methods

A. *Imaging details*

Functional whole-brain scans were performed in interleaved order using T2*-weighted gradient echo-planar imaging (EPI) pulse sequence (TR = 3000 ms; TE = 35 ms; flip angle = 90°, FOV = 220 × 220 mm; 3 mm slice thickness) and included 44 nearly horizontal slices mostly covering the brain. High-resolution T1-weighted anatomical images (voxel size = 1 x 1 x 1 mm) were acquired during the same scanning session using a magnetization-prepared rapid acquisition gradient-echo (MPRAGE) protocol with 176 contiguous slices and the following parameters: TR = 1860 ms; TE = 2.74 ms, flip angle = 8°, FOV = 256 × 256 mm, slice thickness = 1 mm. These anatomical volumes were used for cortical segmentation and surface reconstruction.

B. *MRI Image processing*

Preprocessing of the functional data included 3D-motion correction using trilinear and sinc interpolations, temporal smoothing using a linear trend removal with a high-pass filter (cut-off: 0.01 Hz), and spatial smoothing with a Gaussian filter (6 mm full-width at half-maximum value). The first and the last 20 TRs in each run were removed to eliminate pre-processing artifacts and to allow the hemodynamic responses to reach a steady state. The functional data for each participant were spatially normalized into Talairach space and projected onto a reconstructed cortical surface from the high-resolution 3D anatomical images. All analyses were performed in volume space (by voxel); the figures display activation on the brain surface for illustrative purposes only. All participants exhibited head motion of less than 3 mm (one voxel) in all six directions, three translation parameters, and three rotation parameters, and thus, all participants were included in the fMRI analyses.

C. *Network Cohesion analysis: intra- and inter-NCI*

Intra- and inter-NCI were computed in 10-sample (30 seconds) sliding windows for each participant. We used a nonparametric Wilcoxon rank sum test to examine differences between the PMDD and control groups in each window of interest. The analysis was limited to epochs that were rated previously as evoking sadness at least to a moderate extent. The rating, which ranged from 1 (neutral) to 21 (very deep sadness), was resampled in time windows corresponding to the NCI windows and compared with a null hypothesis of a median rating of 9/21 using Wilcoxon signed-rank test. Thirty-two out of 155 time windows survived this test after FDR correction for multiple dependent comparisons [24]. FDR correction for 32 comparisons was also applied to the results of each family of NCI comparisons in the surviving time windows. Since the overlapping NCI windows are not exclusive, we employed False Discovery Rate (FDR) correction for dependent tests to account for multiple comparisons [24] {Benjamini, 2001 #1850}.

Finally, in case of significant effect, we assessed the spatial specificity of the results, i.e., the extent to which the observed effect is part of a global gray matter effect. We applied a procedure we previously developed to estimate specificity (e.g., [27]), where the original peak coordinates are randomly rotated, translocated, and mirrored in a sampling space. This space included 42,309 gray matter voxels in a mask, demarcated by thresholding ICBM 452 map (http://www.loni.usc.edu/atlases) to exclude voxels with a probability lower than 75% of being classified as gray matter. To generate a null distribution of Z values, we randomized 10,000 networks. For each time window of interest, we compared the Z score resulting from the Wilcoxon test for the difference between the PMDD and control group NCI with the null distribution of Z scores generated for the randomized networks. The specificity index was computed as the percentage of absolute Z values in the null distribution higher or equal to the Z value in the original test.
