# Supplementary material for: Empathy-related abnormalities among women with premenstrual dysphoric disorder: clinical and functional magnetic resonance imaging study
Source: BJPsych Open. 2024 Aug 5;10(5):e138. doi: 10.1192/bjo.2024.723 (PMC11698146; doi:10.1192/bjo.2024.723)

Results for the patient subgroup <u>without</u> pharmacological treatment

1. Inter-SC analysis (N = 18)

Figure S1. The inter-SC analysis was repeated on the subgroup of 18 patients who participated in the fMRI task and who did not receive pharmacological treatment for their psychiatric symptoms. The obtained maps were very close to the maps for all patients. Moreover, a direct comparison between the maps did not reveal any significant findings.

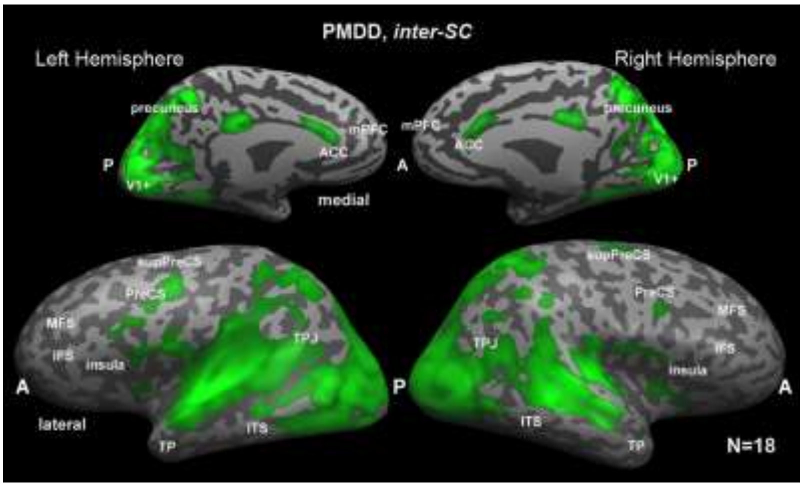

2. **Figure S2.** We have compared the results of IRI for the patient group that did not receive pharmacological treatment with the initial group. To make the groups comparable we randomly excluded six participants from the healthy group and repeated the test twice. The findings are very similar to the outcomes found initially.

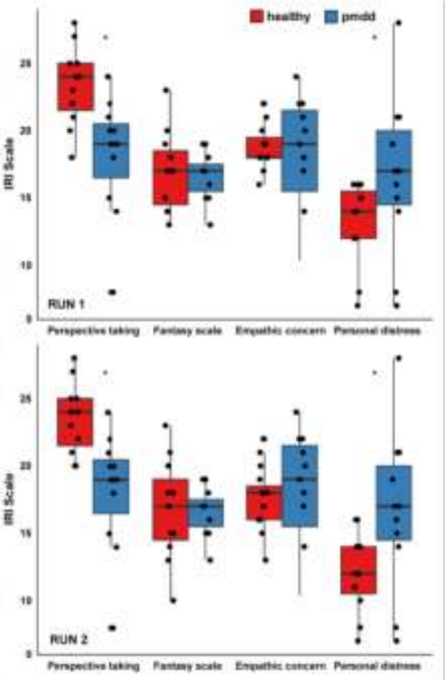

3. Clinical characteristics

**PMTS-OR**	*Depression*	*Anxiety*	*Lability*	*Anger*	*Total*
	3.5 ± 0.6	3.2 ± 0.7	3.4 ± 0.8	3.1 ± 0.6	31.7 ± 4.2
**BFI**	*Extraversion*	*Neuroticism*	*Agreeableness*	*Conscientiousness*	*Openness to experience*
	25.5 ± 6.0	26.3 ± 5.2	34.1 ± 3.9	31.0 ± 4.5	38.3 ± 5.8
**CGI-S**			5.1 ± 0.3		

4. NCI analysis

Figure S3. In the NCI analysis, the statistical results obtained for the ToM-related network did not change after the removal of individuals with PMDD who were treated with medicines.

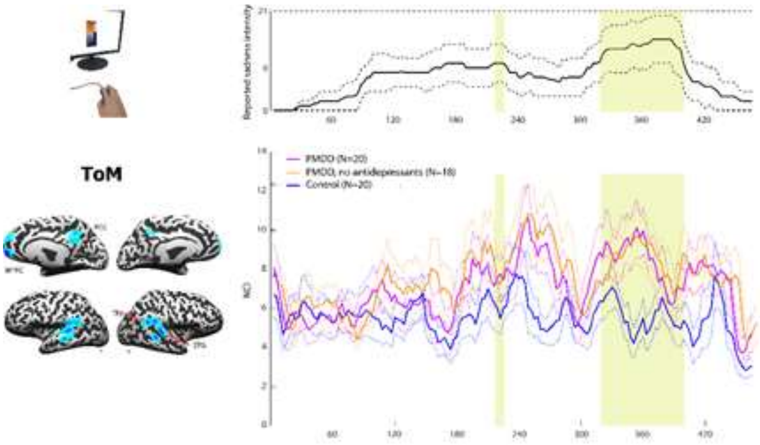

Supplement: Lerner et al. supplementary material 2 — Lerner et al. supplementary material [file S2056472424007233sup002.pdf]
