# Supplementary material for: Empathy-related abnormalities among women with premenstrual dysphoric disorder: clinical and functional magnetic resonance imaging study
Source: BJPsych Open. 2024 Aug 5;10(5):e138. doi: 10.1192/bjo.2024.723 (PMC11698146; doi:10.1192/bjo.2024.723)

Results for the patient subgroups <u>with and without psychological treatment</u>

1. Inter-SC analysis

Figure S4. Twelve patients with PMDD who participated in the fMRI task have received psychological treatment and eight patients have not received it. We repeated the inter-SC analysis on these two subgroups. Figure S6 shows the obtained results; no significant differences were found while comparing the two maps (two-tailed t-test).

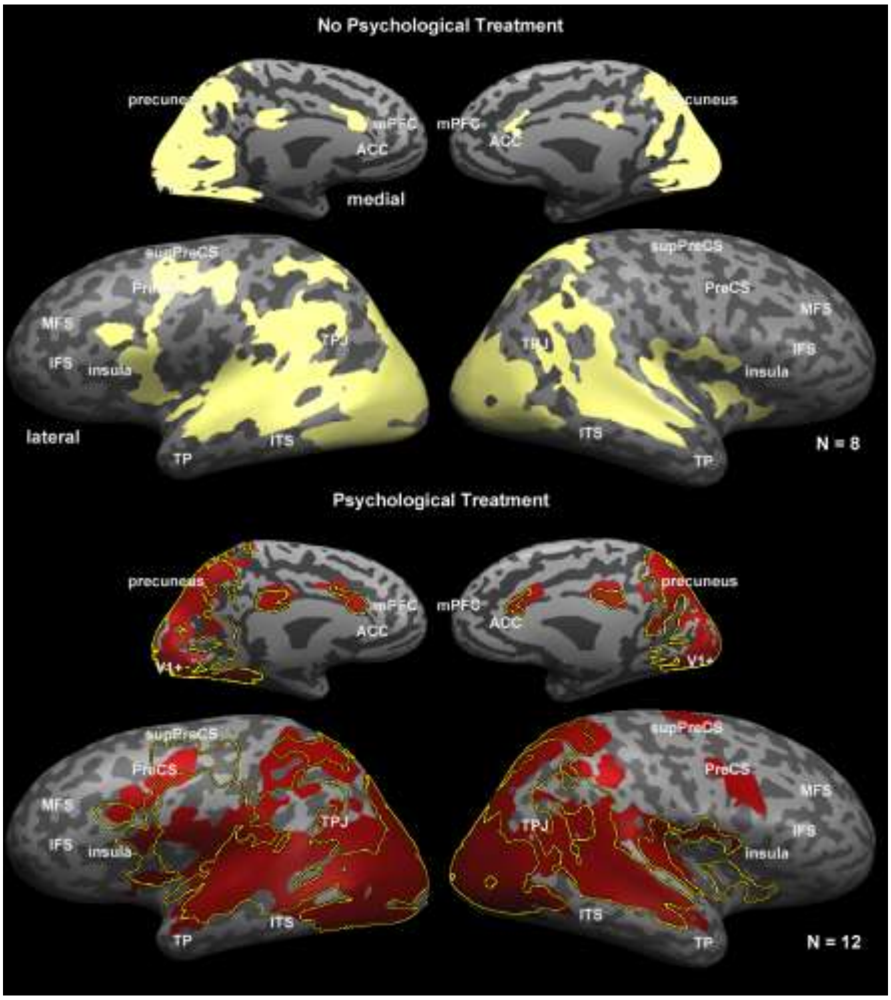

2. Clinical characteristics

PMTS-OR	*Depression*		*Anxiety*		*Lability*		*Anger*		*Total*	
	with	*without*	*with*	*without*	*with*	*without*	*with*	*without*	*with*	*without*
	3.0 ± 1.2	2.8 ± 0.7	2.9 ± 0.9	3.1 ± 0.9	3.4 ± 0.7	2.6 ± 1.5	3.1 ± 0.6	3.0 ± 0.9	29.7 ± 6.7	27.6 ± 9.6

BFI	*Extraversion*		*Neuroticism*		*Agreeableness*		*Conscientiousness*		*Openness to experience*	
	with	*without*	*with*	*without*	*with*	*without*	*with*	*without*	*with*	*without*
	25.4 ± 4.9	25.7 ± 7.7	25.9 ± 6.0	26.8 ± 4.3	33.8 ± 4.5	34.7 ± 2.9	32.7 ± 4.5	28.5 ± 3.4	35.9 ± 4.8	42.0 ± 5.5

CGI-S	*with*	*without*
	4.5 ± 1.0	4.6 ± 1.3

3. **Figure S5**. The ToM NCI did not differ between individuals with PMDD who underwent psychological treatment during the study and those who did not.

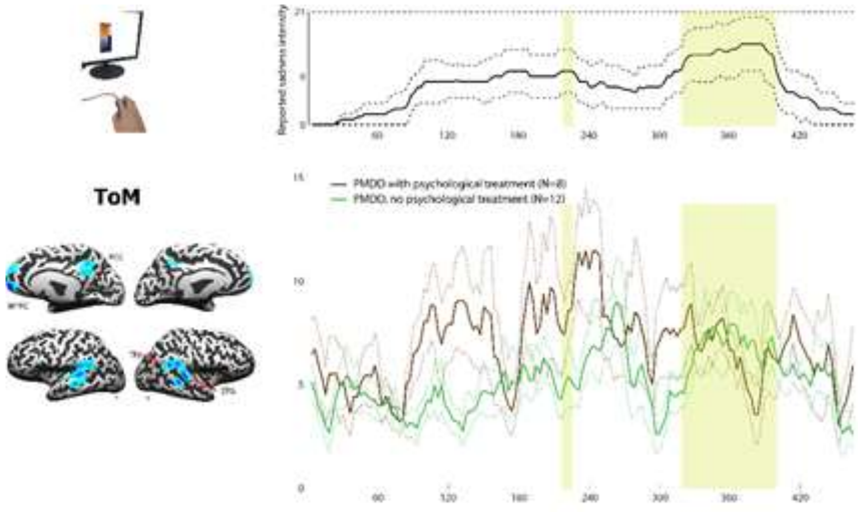

Supplement: Lerner et al. supplementary material 3 — Lerner et al. supplementary material [file S2056472424007233sup003.pdf]
